# The Portuguese version of the screen for disordered eating: validity and reliability in men across multiple ages

**DOI:** 10.1192/j.eurpsy.2024.1173

**Published:** 2024-08-27

**Authors:** A. T. Pereira, A. Silva, M. J. Brito, C. Marques, A. Araújo, A. Macedo, S. Renca

**Affiliations:** ^1^ Institute of Psychological Medicine; ^2^Faculty of Medicine, University of Coimbra, Coimbra, Portugal

## Abstract

**Introduction:**

The Screen for Disordered Eating/SDE was created as a primary care screening method for eating disorders, including binge eating disorder (Maguen et al. 2018). The SDE comprises five items (yes/no answers), extracted from other validated self-reported questionnaires assessing eating psychopathology. Its validity and reliability has proved in a Portuguese psychometric study, that only included woman (Pereira et al. 2022). It psychometric properties have yet to be evaluated in men.

**Objectives:**

We aim to assess the psychometric properties of the Portuguese version of SDE in males.

**Methods:**

Participants were 227 male individuals with a mean age of 30.41 years (±13.96; range: 14-73). They answered an online survey including the Portuguese preliminary versions of the seven-item Eating Disorder Examination Questionnaire/EDE-Q7; the Body Image Concern Inventory/BICI and the Muscle Dysmorphia subscale of the Eating Disorder Assessment for Men/DM-EDAM.

**Results:**

Confirmatory Factor Analysis showed good fit for the unidimensional model (χ^2^/df=1.483; RMSEA=.0460; CFI=.980 TLI=.961, GFI=.988). Cronbach’s alpha was .621 which although inferior to .7 can be explained by the small number of items and the fact that each one assesses different dimensions. All items contributed to the internal consistency and presented high internal validity. Pearson’s correlations of SDE with BICI (.317) and EDE-Q7 (.361) were significant and moderate. The correlation with DM-EDAM was non-significant, probably due to its focus on muscle dysmorphia, which is not included in SDE’s items.

**Conclusions:**

The Portuguese version of SDE demonstrated adequate validity (construct and convergent) and reliability.

**Disclosure of Interest:**

None Declared

